# Health literacy and motivation to change health behavior among cardiovascular patients

**DOI:** 10.1186/s12872-025-04936-w

**Published:** 2025-07-04

**Authors:** Hossein Ghalavand, Abdolahad Nabiolahi, Sirous Panahi

**Affiliations:** 1 Department of Medical library and Information Science, Abadan University of Medical Sciences, Abadan, Iran; 2 Department of Medical library and Information Science, School of Allied Medical Sciences, Zahedan University of Medical Sciences, Zahedan, Iran; 3 Department of Medical library and Information Science, School of Health Management and Information Sciences, Iran University of Medical Sciences, Tehran, Iran

**Keywords:** Health literacy, Health behavior, Motivation, Cardiovascular abnormalities, Patients

## Abstract

**Introduction:**

Today, cardiovascular diseases (CVDs) have become increasingly prevalent in various societies. Having an adequate level of health literacy and necessary motivation to modify high-risk behaviors are among the key factors influencing the improvement of patient conditions. The present study investigates the relationship between health literacy and motivation to change health behavior among patients with CVDs.

**Methodology:**

This is a cross-sectional, correlational research. The research population consists of 190 patients with CVDs and a history of hospitalization, who are randomly selected. Health literacy questionnaire and Health Behavior Motivation Scale were used for data collection. The data were analyzed using SPSS 26.0, and descriptive statistics of mean, standard deviation and Pearson’s correlation coefficient.

**Results:**

189 cardiovascular patients participated in this study. The mean health literacy scores of patients with CVDs were 23.91, 15.42, 27.38, 15.59 and 46.39 in terms of access, reading skills, understanding, appraisal and application of health information, respectively. Scores for motivation to change health behaviors between CVDs patients revealed means for autonomous motivation were 3.67, for controlled motivation stands at 3.48, and for the probability rating assigned by physicians were 4.06. Considering the significance level of Sig > 0.05, except for understanding health information (f = 2.962, Sig = 0.021), no significant correlation was found between health literacy dimensions and patients’ age. The age variable was not correlated with any of the dimensions of motivation to change patients’ behavior. Moreover, no significant difference was observed between patients’ educational level and their health literacy and motivation for change health behavior dimensions scores (Sig > 0.05). There was no significant relationship between sex and dimensions of health literacy and motivation for health behavior change (Sig > 0.05). Furthermore, a significant correlation was found between patients’ scores in five dimensions of health literacy and motivation for health behavior change (*r* = 0.154, Sig = 0.034).

**Conclusion:**

Considering the undesirable level of health literacy among patients with CVDs and a strong correlation between levels of health literacy and motivation for health behavior change, it is recommended that health literacy improvement programs must be developed by health-related institutions through various innovative educational interventions in different in-person and online settings.

**Supplementary Information:**

The online version contains supplementary material available at 10.1186/s12872-025-04936-w.

## Introduction

Cardiovascular diseases (CVDs) are the leading cause of mortality worldwide [1–4]. The total number of deaths related to CVDs is estimated to be 17.9 million annually. Several factors, such as blood pressure, blood lipids, hyperglycemia and obesity, are the primary contributors to CVDs [5]. These conditions could also be associated with an unhealthy lifestyle [6, 7]. Approximately 40–60% of the risk for coronary artery disease is attributed to genetic predisposition [5]. Smoking, alcohol consumption, unhealthy diets, stress, obesity, high cholesterol, hypertension and diabetes are significant risk factors for coronary artery diseases and powerful predictors of changes in disease prevalence among populations [3, 8]. In this regard, having adequate health literacy and necessary motivation to change behaviors related to risk factors are critical factors for improving patient conditions [9–11].

Having the motivation to change health behaviors is a significant challenge for patients [12]. Behavior change is difficult for individuals who lack the motivation to engage in health-related behaviors. Conscious attention to the benefits and drawbacks are the two main processes involved in behavior change [13]. Many unhealthy behaviors, such as poor eating habits, smoking and alcohol consumption, are associated with n and reward-based outcomes, which are typically governed by dopaminergic pathways in the brain that act as strong reinforces of behavior [14]. Thus, exerting conscious control to overcome these strong neural associations between cues and actions is challenging and requires significant cognitive resources and motivation [15]. Individuals who lack motivation to engage in health-related behaviors are unlikely to change, and patients with low motivation for self-care are less likely to adhere to a healthy lifestyle [16, 17].

Health literacy, recognized as a significant factor influencing adherence to healthcare and health behaviors, is a powerful tool for prevention and patient education [18]. Therefore, it has recently gained attention in the management of chronic diseases, particularly CVDs [19, 20]. Conceptually, health literacy could be defined as the ability to read, understand and act upon, listen, apply, analyze and make decisions based on health and medical recommendations [10, 21]. According to World Health Organization (WHO), health literacy is defined as cognitive and social skills that determine one’s motivation and ability to access, understand and apply information in a way that promote and maintain their health. WHO emphasized that health literacy should not only be regarded as an individual trait, but also as a key determinant of community health [10]. Health literacy enables individuals to comprehend medication guides, patient consent forms, home care manuals and details of healthcare programs [20]. Individuals with low health literacy are less likely to understand written and verbal information provided by healthcare professionals and are less likely to follow given instructions [10]. Lack of information about the disease could affect patients’ self-confidence or self-efficacy [22].

Patients with CVDs need to manage their condition, medications, fluid intake, physical activity and symptoms, and follow up their self-care through consultations and visits with healthcare providers. Adherence to healthcare providers’ recommendations and self-care regimens requires applying educational initiatives, principles and knowledge to make care-related decisions and manage conditions. Even if patients with CVDs receive adequate education regarding self-management based on clinical guidelines, insufficient health literacy could be a potential barrier to acquiring the necessary knowledge and self-management skills [3]. Illiterate individuals have lower awareness of their condition, which negatively impacts their quality of life [23]. Moreover, inadequate health literacy has been reported as a significant factor contributing to reduced self-care behaviors and worsening of health status among patients with CVDs [24].

Investigating the relationship between health literacy and hospital readmission among patients with myocardial infarction at four teaching hospitals in Tehran revealed a significant correlation between health literacy and readmission rate [25]. Additionally, a significantly positive correlation was found between the mean scores of self-care and health literacy [11]. In a study, analyzing the health literacy of patients with a history of open-heart surgery indicated these patients did not have optimal health literacy [26]. In another research, no significant correlation was observed between health literacy and medication adherence among Chinese patients. However, regression models revealed patients with limited health literacy were significantly more likely to fail to adhere to a heart-healthy lifestyle [27].

Based on the provided text, a potential scientific research gap could be the need for further investigation into the impact of health literacy on medical recommendation and lifestyle choices among CVDs patients. While some studies have highlighted the correlation between health literacy and self-care behaviors [28–30], there remains a gap in understanding the specific relationship between health literacy levels and motivation for change inappropriate health behavior among individuals with CVDs. Previous studies have mainly focused on inadequate health literacy, and the relationship between different levels of health literacy and motivation for behavior change among patients with CVDs has received less attention [7, 31, 32]. Therefore, the present study aims to investigate the relationship between health literacy and motivation for health behavior change among patients with CVDs. Understanding the relationship between health literacy and motivation for behavior change could provide opportunities for developing the necessary interventions to improve health literacy and facilitate the modification of unhealthy behaviors among cardiovascular patients. By conducting research in this area, healthcare professionals and policymakers can gain valuable insights into the role of health literacy in promoting better outcomes and quality of life for individuals living with cardiovascular diseases. This research aims to achieve the following objectives:

1. Determining the mean scores for health literacy among patients with CVDs.
2. Determining the mean score for health behavior change among patients with CVDs.
3. Determining the relationship between health literacy and motivation for behavior change among patients with CVDs.

## Methodology

This study was a quantitative, correlational and cross-sectional work. The population consisted of patients with CVDs and a history of hospitalization at Ayatollah Taleghani Hospital in Abadan, Iran. The inclusion criteria were diagnosis of one of the cardiovascular diseases by a cardiologist, absence of other underlying conditions, being in the age range of 18–65 years old and being willing to participate in the study. Individuals who were not able to read or had congenital cardiovascular diseases were excluded from the study. Since the beginning of 2023, a total of 321 patients have been admitted and discharged from Ayatollah Taleghani Hospital, 190 of whom were randomly selected using Morgan’s table. Patients’ data were retrieved from their medical records stored in the hospitals. Each patient was assigned a unique numerical code, and a final sequential list was prepared and used for sampling. A simple random sampling method was used to ensure that each patient had an equal chance of being selected. Based on unique numerical codes, patients were selected using a random number table. Patients’ data were subsequently retrieved from their medical records stored in the hospital.

In this study, two questionnaires has previously been published were used to collect data. First, Data were collected using Health Literacy for Iranian Adults (HELIA) questionnaire [33]. This questionnaire consists of five dimension and 33 items for health literacy, scored based on a Likert scale (ranging from 1 = never to 5 = always). Dimensions of health literacy in HELIA include access to health information (Q1-Q6), reading skills of health information (Q7-Q10), understanding health information (Q11-Q17), appraisal health information (Q18-Q21) and application of health information (Q12-Q33) (Appendix A). Its validity and reliability have been repeatedly confirmed in previous studies [3, 26].

Second, Health Behavior Motivation Scale, developed by Williams et al. (2005), was employed [34]. This scale includes three dimension and 12 items, scored based on a 5-point Likert scale (ranging from 1 = strongly agree to 5 = strongly disagree). Autonomous motivation (Q1-Q4), controlled motivation (Q5-Q7) and perceived support from the physician (Q8-Q12) are dimensions for motivation to change behavior (Appendix B). The internal consistency of the whole scale was obtained as 0.89 using Cronbach’s alpha [34]. Various studies have confirmed its content and construct validity. Its reliability was reported as 0.79 using Cronbach’s alpha [1].

In this research, a systematic methodology was implemented to address the issue of missing data. Initially, the characteristics and magnitude of the missing data were evaluated to categorize it as missing completely at random, missing at random, or missing not at random. To manage the missing values, multiple imputation techniques were utilized. This approach facilitates the integration of the uncertainty linked to the missing values, thereby improving the robustness of the statistical analyses [35, 36]. Furthermore, sensitivity analyses were performed to assess the influence of the imputation strategy on the overall findings. These analyses involved comparing results obtained from imputed datasets with those derived from complete-case analyses, ensuring that the conclusions remained consistent and reliable in the presence of missing data.

### Data analysis

Data analysis was conducted using SPSS 26.0.The relationship between the variables under study was assessed using descriptive statistics, including mean and standard deviation. Additionally, analytical statistic, including Pearson’s correlation coefficient at the significance level of 0.05, was used. Normal distribution of the data was assessed using Shapiro-Wilk test, and the results indicated the data were normally distributed.

## Results

Out of 189 patients with CVDs, 57.1% were female and 42.9% were male (Table 1). In terms of age, the lowest proportion of participants were in the age group of 18–28 years, while the highest proportion (34.9%) were aged over 60 years old. Regarding educational level, the majority of participants (44.4%) had a high school diploma.

**Table 1:** Characteristics of the study group

Variables	Categories of Variable	number	percent
Gender	Male	81	42.9
	Female	108	57.1
	Total	189	100
Age	18–28	19	10.1
	29–39	24	12.7
	40–49	32	16.9
	50–60	48	25.4
	More than 60	166	34.9
Education	Less than Diploma	64	33.9
	Diploma	84	44.4
	Bachelor and above	40	21.2
	Total	189	100

The results in Table 2 indicates that the mean scores of health literacy were obtained as 23.91, 15.42, 27.38, 15.59 and 46.39 in terms of access, reading skills, understanding, appraisal and application of health information, respectively. In addition, the mean scores of the motivation for changing health behaviors among cardiovascular patients indicated an autonomous motivation for change average is 3.67 a controlled motivation for change is 3.48, and physician-rated probability is 4.06.

**Table 2:** Health literacy and motivation to change behavior among CVDs patients

Variables	Dimensions	*N*	Mean	St.D
Health literacy	Access to health information	189	23.91	4.70
	Reading skills of health information	189	15.42	3.39
	Understanding health information	189	27.38	5.60
	Appraisal health information	189	15.59	2.76
	Application of health information	189	46.39	6.18
	Total	189	128.70	20.22
Motivation to change health behavior	Autonomous motivation	189	3.67	0.74
	Controlled motivation	189	3.48	0.89
	Perceived support from the physician	189	4.06	0.41
	Total	189	11.23	1.52

As presented in Table 3 regarding the correlation between patients’ age, health literacy dimensions and motivation to change behavior, the ANOVA results revealed no significant correlation between CVDs patients’ age and dimensions of access to access to health information, reading skills of health information, understanding health information, appraisal health information and application of health information considering the significance level of Sig > 0.05. However, a significant correlation was observed between patients’ age and understanding health information (Sig < 0.05). Moreover, the study found that there were no significant differences between the ages of the cardiovascular patients and dimensions of motivation to change behaviors. This includes autonomous motivation, controlled motivation and perceived support from the physician, all of which had significance values (Sig > 0.05).

**Table 3:** Age, health literacy and motivation to change behavior among CVDs patients

Variables	Dimensions	Groups	Sum of Squares	df	Mean Squares	f	sig
Health literacy	Access to health information	1	162.700	4	40.670	1.800	0.117
		2	3994.767	184	21.711	-	-
		T	4157.471	188	-	-	-
	Reading skill of health information	1	76.209	4	19.050	1.670	0.157
		2	2092.076	184	11.370	-	-
		T	2168.286	188	-	-	-
	Understanding health information	1	357.570	4	89.393	2.962	0.021
		2	5553.231	184	27.380	-	-
		T	5910.804	188	-	-	-
	Appraisal health information	1	47.544	4	11.886	1.569	0.184
		2	139.080	184	7.570	-	-
		T	1441.600	188	-	-	-
	Application of health information	1	427.220	4	106.800	2.090	0.230
		2	6771.700	184	36.803	-	-
		T	7199.020	188	-	-	-
Motivation to change behavior	Autonomous motivation	1	10.921	7	1.560	0.863	0.537
		2	327.407	181	1.809	-	-
		T	338.328	188	-	-	-
	Controlled motivation	1	8.443	5	1.689	0.937	0.450
		2	329.880	183	1.803	-	-
		T	338.320	188	-	-	-
	Perceived support from the physician	1	8.618	2	4.309	2.430	0.910
		2	329.710	186	1.773	-	-
		T	338.328	188	-	-	-

1 = Between groups 2 = Within groups T = Total

As presented in Table 4 regarding the relationship between patients’ educational level, health literacy and motivation to change behavior, the ANOVA results showed no significant correlation between patients’ educational level and five dimensions of health literacy (*p* > 0.05). The study also demonstrated that there were no substantial differences in the educational attainment of patients concerning their motivation to change behavior (*p* > 0.05).

**Table 4:** Education level, health literacy and motivation to change behavior among CVDs patients

Variables	Dimensions	Groups	Sum of Squares	df	Mean Squares	f	sig
Health literacy	Access to health information	1	7.790	2	3.800	0.174	0.841
		2	4149.600	185	22.400	-	-
		T	4157.400	187	-	-	-
	Reading skill of health information	1	0.867	2	0.434	0.037	0.964
		2	2164.936	185	11.700	-	-
		T	2165.800	187	-	-	-
	Understanding health information	1	26.850	2	13.420	0.420	0.650
		2	5842.950	185	31.580	-	-
		T	5869.800	187	-	-	-
	Appraisal health information	1	44.615	2	22.307	2.950	0.055
		2	1396.660	185	7.550	-	-
		T	1441.270	187	-	-	-
	Application of health information	1	82.308	2	41.150	1.080	0.342
		2	7045.920	185	38.086	-	-
		T	7128.230	187	-	-	-
Motivation to change behavior	Autonomous motivation	1	2.825	7	0.404	0.741	0.638
		2	98.110	180	0.454	-	-
		T	100.930	187	-	-	-
	Controlled motivation	1	1.751	5	0.350	0.643	0.667
		2	99.180	182	0.540	-	-
		T	100.930	187	-	-	-
	Perceived support from the physician	1	1.268	2	0.634	1.177	0.310
		2	99.660	185	0.530	-	-
		T	100.930	187	-	-	-

1 = Between groups 2 = Within groups T = Total

Moreover, there was no significant difference in the mean scores of five dimensions of health literacy between men and women. Alternatively, the findings indicated that the average scores across the motivation to change behavior did not differ significantly between the groups of women and men (Table 5).

**Table 5:** Sex, health literacy and motivation to change behavior among CVDs patients

Variables	Dimensions	Sex	*N*	Mean ± St.D	df	Sig
Health literacy	Access to health information	F	81	23.90 ± 5.07	187	0.094
		M	108	23.91 ± 4.42		
	Reading skill of health information	F	81	15.34 ± 3.50	187	0.572
		M	108	15.49 ± 3.32		
	Understanding health information	F	81	27.24 ± 6.07	187	0.279
		M	108	27.49 ± 5.25		
	Appraisal health information	F	81	15.85 ± 2.77	187	0.873
		M	108	15.39 ± 2.76		
	Application of health information	F	81	46.60 ± 6.34	187	0.883
		M	108	46.23 ± 6.09		
Motivation to change behavior	Autonomous motivation	F	81	3.80 ± 0.74	187	0.613
		M	108	3.58 ± 0.73		
	Controlled motivation	F	81	3.58 ± 0.98	187	0.115
		M	108	3.40 ± 0.80		
	Perceived support from the physician	F	81	4.074 ± 0.38	187	0.471
		M	108	4.06 ± 0.43		

Pearson’s correlation coefficient was used to assess the correlation between health literacy and motivation for behavior change among patients with CVDs. As presented in Table 6, the analysis revealed that there was no substantial correlation between the “access to health information” and the dimension of motivation for behavioral change, as indicated by the reliability coefficient. However, a significant relationship was identified between the “perceived support from the physician” in fostering independence and the motivation to change behavior in relation to the “access to health information”. In examining the five dimensions of health literacy, the study found no significant associations between the reading skills, understanding, and appraisal of health information and the three dimension of motivation to change behavior. Overall, the data presented in Table 6 indicates a substantial correlation between the motivation for behavioral change among cardiovascular patients and their health literacy, as determined by the confidence coefficient.

**Table 6:** Relationship between health literacy and motivation to change behavior among CVDs patients

Dimensions	Autonomous motivation		Controlled motivation		Perceived support from the physician		Motivation to change behavior			
	*r*	sig	*r*	sig	*r*	sig	*r*	95% CI	sig	Effect Size
Access to health information	0.095	0.192	0.031	0.675	0.345	< 0.001	0.158	23.23–24.58	0.030	Small
Reading skill of health information	0.090	0.219	0.00	1.000	0.312	< 0.001	0.129	14.92–15.91	0.078	Small
Understanding health information	0.082	0.265	0.04	0.552	0.200	0.006	0.119	26.58–28.23	0.101	Small
Appraisal health information	0.024	0.743	−0.06	0.410	0.229	0.002	0.039	15.21–15.98	0.597	Negligible
Application of health information	0.145	0.046	0.07	0.340	0.281	< 0.001	0.188	45.50–47.29	0.010	Small
Health literacy	0.108	0.14	0.03	0.659	0.305	< 0.001	0.154	125.89–131.52	0.034	Small

## Discussion

Nowadays, chronic diseases are recognized as the leading cause of mortality, accounting for 74% of deaths in the general population. 86% of deaths occur before the age of 70, particularly in low-income countries. Major risk factors contributing to the spread of these diseases include tobacco use, excessive alcohol consumption, physical inactivity, poor diet and air pollution [37]. Given the impact of various factors, changing individuals’ lifestyle in society, creating motivation for health behavior change and promoting health literacy have become inevitable. Therefore, this study was conducted to investigate the relationship between health literacy levels and motivation for health behavior change among patients with CVDs.

Assessing health literacy levels among patients with CVDs revealed the mean scores of health literacy as 23.91, 15.42, 27.38, 15.59 and 46.39 in dimensions of access, reading skills, understanding, appraisal and application of health information, respectively. The highest score of health literacy was related to “application of health information”. In line with the present research, previous studies conducted in Iran have indicated that health literacy of patients with CVDs was at the borderline/moderate level [3, 38, 39]. However, inconsistent with our work, some studies conducted in other countries have indicated the health literacy level of patients with CVDs was significantly higher [40–43].

The results showed no significant correlation between patients’ age and educational level and health literacy dimensions (access to health information, reading skill of health information, health information appraisal and application of health information) (Sig > 0.05). However, a significant correlation was found between patients’ age and understanding health information (Sig < 0.05). Inconsistent with our investigation, some studies have revealed suffering from CVDs is independently associated with inadequate health literacy considering factors such as age, educational level, income, health awareness and social support [40, 41]. Additionally, a statistically significant correlation was found between CVDs patients’ health literacy level, their age and educational level [44].

The regression analysis results showed a significant correlation between health literacy dimensions (access to health information, reading skill of health information, understanding health information and application of health information) and motivation for health behavior change among CVD patients (*p* < 0.001). However, no significant correlation was found between health information appraisal and motivation for behavior change (*p* < 0.027). In line with our work, previous studies reported an insignificant positive correlation between patient activities and self-care behaviors among patients with heart failure [9, 45, 46]. Also, studies conducted using the theory of planned behavior and health literacy and health action framework have shown patients often report numerous unmet needs upon discharge. Implementing tailored approaches for health literacy requires providing an environment supported by a multi-faceted approach, particularly one that involves changing individual behaviors and engaging patients and their families [41, 43].

In the present study, a significant correlation was observed between patients’ health literacy level and their motivation for health behavior change. Analyzing the relationship between health literacy and adherence to preventive behaviors among patients with CAD indicated they had limited health literacy. Moreover, no significant correlation was found between health literacy and medication adherence. However, the regression analysis results revealed a significant correlation between health literacy and non-adherence to a healthy lifestyle [27, 47]. Other researchers have pointed out that inadequate health literacy is strongly associated with mortality, complications and use of healthcare services [11, 25, 26, 39, 43, 45, 47]. The American Heart Association emphasized the importance of the relationship between health literacy and CVDs and outlined five appropriate actions to clarify the relationship between health literacy and heart health, including adverse impact of poor health literacy on risk factors, conditions and treatment, development of appropriate health literacy strategies to prevent diseases, identifying the relationship between social determinants and providing useful guidelines for future advancements in treatment and research on CVDs [4, 7]. In the current study, patients emphasized the importance of having options provided by physicians to facilitate health behavior changes, which requires greater attention from the healthcare professionals.

The implications of non-significant findings in current study on health literacy and motivation for health behavior change among CVDs patients are multifaceted. Non-significant correlations, such as those between patients’ age and educational level with certain health literacy dimensions, do not necessarily indicate a lack of effect but might reflect limitations in statistical power or sample size. This can lead to misinterpretation and missed opportunities for targeted interventions. Furthermore, potential confounding factors like socioeconomic status, access to healthcare, and cultural beliefs can influence both health literacy and behavior change, necessitating careful control in study design and analysis to ensure accurate conclusions. Addressing these factors is crucial for developing effective strategies to enhance health literacy and promote health behavior change among CVDs patients.

The significant correlation between health literacy and motivation for health behavior change underscores the importance of enhancing health literacy through tailored educational programs. Healthcare providers can use these insights to develop targeted interventions that improve patients’ ability to access, understand, and apply health information, thereby fostering greater motivation for lifestyle changes. Additionally, the emphasis on providing options by physicians to facilitate health behavior changes highlights the need for patient-centered care approaches that engage both patients and their families in the decision-making process. By implementing these strategies, healthcare systems can better support patients in managing chronic conditions, reducing complications, and improving overall quality of life.

This study had several limitations. The research predominantly utilized univariate statistical methods, which may inadequately reflect the intricate relationships between health literacy and various health behaviors. The cross-sectional design of the study restricts the capacity to determine temporal relationships between health literacy and health outcomes. Employing longitudinal methodologies would yield stronger evidence regarding causality. The analysis may overlook potential moderators or mediators that could affect the identified relationships. Patients of a single cultural and social context participated in this study, and the generalization of the results is very difficult and limited. The dependence on self-reported data for health behaviors and literacy may introduce bias. Utilizing objective measures or mixed-methods approaches could yield more precise evaluations. Although the study underscores the significance of health literacy, it lacks specific strategies for enhancing literacy or health behaviors. Finally, an electronic questionnaire designed for data collection was used. Some patients lacked the necessary tools, such as smartphones, personal computers, etc., to access this questionnaire and faced limitations in internet-based communication.

## Conclusion

The results of current research indicated a significant correlation between the motivation for health behavior change and health literacy among patients with CVDs. Therefore, it is recommended that programs aimed at developing health literacy, particularly regarding risk factors for CDVs, be prioritized by health policymakers in order to facilitate lifestyle changes and enhance patient motivation. Furthermore, it would be appropriate to include topics that promote desirable health behaviors and enhance health literacy in health education programs. Policymakers should integrate health literacy into healthcare systems by mandating clear language in patient materials and training healthcare providers in effective communication. Public health campaigns can also focus on improving health literacy, particularly in low-income communities. Practitioners can implement tailored interventions using visual aids and simple language, and encourage patient engagement through shared decision-making.

## Supplementary Information

Supplementary Material 1

Supplementary Material 2

## Data Availability

The datasets used and analysed during the current study are available from the corresponding author on reasonable request.

## Abbreviations

CVDs: Cardiovascular Diseases
HELIA: Health Literacy for Iranian Adults
