# Supplementary material for: Health literacy and motivation to change health behavior among cardiovascular patients
Source: BMC Cardiovasc Disord. 2025 Jul 4;25:479. doi: 10.1186/s12872-025-04936-w (PMC12231721; doi:10.1186/s12872-025-04936-w)
Supplement: Supplementary file 1 — Supplementary Material 1 [file 12872_2025_4936_MOESM1_ESM.pdf]

Health Literacy for Iranian Adults

(HELIA)

Access to health information

Q1: I can obtain the health and medical information I need from various sources.

Q2: I can obtain information about healthy nutrition.

Q3: I can obtain information about mental health such as depression and stress.

Q4: I can obtain information about the disease I want to know.

Q5: I can obtain the information I need about some health problems and diseases such as high blood pressure, as well as high blood sugar and fat.

Q6: I can obtain information about the harms and risks of smoking.

Reading skills of health information

Q7: It is easy for me to read educational materials about health and wellness (booklets, pamphlets, educational and promotional brochures).

Q8: It is easy for me to read specific written instructions that doctors, dentists and health workers give me about my illness.

Q9: It is easy for me to read medical and dental forms (such as patient admission forms, consent forms, file formation, etc. in hospitals and medical centers).

Q10: It is easy for me to read instructions and preparation sheets before performing an examination, ultrasound or radiology.

Understanding health information

Q11: I understand recommendations for healthy eating.

Q12: I understand the explanations that the doctor gives about my illness.

Q13: I understand the meaning and significance of the contents written on medical and dental forms (such as patient admission forms, consent forms, file formation, etc. in hospitals and medical centers).

Q14: I understand the meaning and significance of the signs and contents written on signboards in hospitals, clinics, and health centers.

Q15: I understand the instructions for taking medication written on the medication packaging.

Q16: I understand the advantages and disadvantages of the treatment methods prescribed by the doctor.

Q17: I understand the meaning and significance of the contents written on the instruction sheet before performing an examination, ultrasound, or radiology.

Appraisal health information

Q18: I can evaluate the accuracy of health-related information provided on the Internet.

Q19: I can evaluate the accuracy of health information provided on television and radio.

Q20: I can evaluate the accuracy of health advice given to me by friends and relatives.

Q21: I can pass on health information I have learned to others.

Application of health information

Q22: I know where or who to go to when I see the symptoms of an illness.

Q23: When the doctor recommends "take an antibiotic capsule three times a day, on time," I follow the 8-hour interval.

Q24: I do not stop taking the medications my doctor has prescribed for my illness without his permission, even if the symptoms have disappeared.

Q25: If one or more of my first-degree relatives have been diagnosed with certain cancers (such as prostate, breast, cervical, and colon cancer, etc.), I will see a doctor for a checkup.

Q26: I will avoid doing things or consuming substances that increase blood pressure.

Q27: I will see a doctor for a checkup (periodic examination) every year, even if I have no symptoms of the disease.

Q28: I will take care of my health in every job and situation.

Q29: If I have any questions about my illness, I will ask the medical and health-care staff.

Q30: I will buy dairy products (milk, yogurt, cheese, etc.) according to the fat content.

Q31: I will avoid doing things or consuming substances that cause weight gain.

Q32: I will wear a seat belt while driving.

Q33: When buying food, I will pay attention to its nutritional value.
