# Supplementary material for: Health literacy and motivation to change health behavior among cardiovascular patients
Source: BMC Cardiovasc Disord. 2025 Jul 4;25:479. doi: 10.1186/s12872-025-04936-w (PMC12231721; doi:10.1186/s12872-025-04936-w)
Supplement: Supplementary file 2 — Supplementary Material 2 [file 12872_2025_4936_MOESM2_ESM.pdf]

Health Behavior Motivation Scale

I will make the recommended lifestyle changes (such as exercising more, reducing fat and salt in my diet, reducing stress, not smoking, etc.) because:

Autonomous motivation

Q1. staying healthy as much as I can is a personal challenge.

Q2. I believe that making such changes will improve my health.

Q3. I feel that changing my lifestyle is a good way to improve my health.

Q4. It is exciting for me to keep myself healthier.

Controlled motivation

Q5. I feel guilty if I don't tell my doctor what I need to.

Q6. I want my doctor to think I am a good patient.

Q7. I feel bad if I don't tell my doctor about my health.

Perceived support from the physician

Q8. I feel that my doctor provides me with options and choices.

Q9. I feel that my doctor understands me.

Q10. My doctor trusts me in my ability to make changes.

Q11. My doctor encourages me to ask questions.

Q12. My doctor tries to understand my views before suggesting new ways of doing things.
